# Recent Advances in Delivery Systems and Therapeutics of Cinnarizine: A Poorly Water Soluble Drug with Absorption Window in Stomach

**DOI:** 10.1155/2014/479246

**Published:** 2014-11-13

**Authors:** Smita Raghuvanshi, Kamla Pathak

**Affiliations:** Department of Pharmaceutics, Rajiv Academy for Pharmacy, N.H. No. 2, P.O. Chattikara, Mathura, Uttar Pradesh 281001, India

## Abstract

Low solubility causing low dissolution in gastrointestinal tract is the major problem for drugs meant for systemic action after oral administration, like cinnarizine. Pharmaceutical products of cinnarizine are commercialized globally as immediate release preparations presenting low absorption with low and erratic bioavailability. Approaches to enhance bioavailability are widely cited in the literature. An attempt has been made to review the bioavailability complications and clinical therapeutics of poorly water soluble drug: cinnarizine. The interest of writing this paper is to summarize the pharmacokinetic limitations of drug with special focus on strategies to improvise bioavailability along with effectiveness of novel dosage forms to circumvent the obstacle. The paper provides insight to the approaches to overcome low and erratic bioavailability of cinnarizine by cyclodextrin complexes and novel dosage forms: self-nanoemulsifying systems and buoyant microparticulates. Nanoformulations need to systematically explored in future, for their new clinical role in prophylaxis of migraine attacks in children. Clinical reports have affirmed the role of cinnarizine in migraine prophylaxis. Research needs to be dedicated to develop dosage forms for efficacious bioavailability and drug directly to brain.

## 1. Introduction

Low aqueous solubility of drug has always presented major obstacle towards the development of drug delivery systems which often compromises patient compliance. Oral route is thought to be common and easy for drug administration. On oral administration of drug in its dosage form, it is expected to dissolve and release the drug into the gastrointestinal fluid before the absorption [1]. Poor solubility may limit the dissolution of drug in gastrointestinal tract resulting to low bioavailability that can pharmacologically affect the therapeutic efficacy of drug [2]. The drugs belonging to BCS class II and IV particularly fall in this category and have been extensively researched molecular optimization and development of novel efficacious dosage forms.

**Figure 1:**
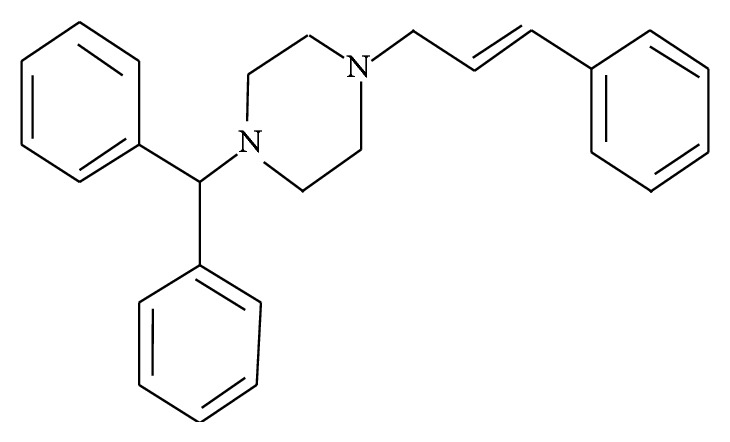
Structure of cinnarizine.

Cinnarizine, (E)-1-(diphenylmethyl)-4-(3-phenylprop-2-enyl) piperazine (Figure 1), molecular formula: C_26_H_28_N_2_ and molecular weight: 368.51 g/mol, is white or almost white powder. Originally obtained from woodreed roots (*Cinna*), cinnarizine was first synthesized by Janssen Pharmaceutica in 1955 and marketed in 1958 under the brand name Stugeron. It is a weak base with poor aqueous solubility. According to a paper the solubility of cinnarizine is highly pH dependent, that is, 0.29 mg/mL at pH 2, 0.017 mg/mL at pH 5, and 0.002 mg/mL at high pH of 6.5 (all the results were at 37°C) [3]. It is highly lipophilic molecule with log⁡*P* value 5.71 [4]. Pharmaceutically it belongs to BCS class II, as displays preferential absorption in stomach (Table 1).

### 1.1. Pharmacokinetics

After oral administration, cinnarizine is rapidly absorbed (*T*_max⁡_ = 2–4 h) from gastrointestinal tract, with an absorption window in upper gastrointestinal tract [5]. On repetitive dosing in healthy human, accumulation of cinnarizine occurs due to its pharmacokinetic properties [6]. The drug can cross the blood-brain barrier by simple diffusion, due to weakly basic and lipophilic [7]. It is metabolized preferably in liver by glucuronidation and oxidation. Cinnarizine is oxidized by cytochrome P450 into metabolites such as 1-(diphenylmethyl)piperazine(C1), 1(diphenylmethyl)-4-[3-(4′-hydroxyphenyl)-2-propenyl] piperazine (C2), and benzophenone(c3) 1-[(4′-hydroxyphenyl)-phenylmethyl]4-(3-phenyl-2-propenyl)piperazine(C4) [8]. Among the various CYP enzymes, only CYP2D6 can catalyze p-hydroxylation of the cinnamyl phenyl ring of cinnarizine (C-2 formation) and only CYP2B6 can catalyze p-hydroxylation of the diphenylmethyl group (C-4 formation) of cinnarizine. On the other hand, CYP2C9 with CYP1A1, -1A2, and/or -2A6 have affinity for N-desalkylation at the 1- and 4-positions of the piperazine ring that results in C-1 and C-3 formation. These promoted microsomal metabolism of cinnarizine [9, 10]. It has high protein binding capacity of 91% [11]. The drug is predominantly excreted in urine as metabolites and in feces mainly as unchanged drug with elimination half-life of 3–6 h. The pharmacokinetic aspects are also compiled in Table 1.

### 1.2. Pharmacodynamics

Pharmacodynamically, cinnarizine is classified as an antihistamine and calcium channel blocker. Additionally, it also shows anticholinergic, antiserotonergic, and antidopaminergic activity [12]. Cinnarizine is moderately sedative antihistaminic agent that acts by inhibition of H1 receptor. It is selective calcium ion entry blocker, which selectively acts on arteries. These agents do not show action on slow calcium channels in myocardium but have selective action on arteries. The drug binds to target calcium channels when they are in an open conformation [13]. The inhibitory effects of cinnarizine on the noradrenaline and Ca-induced contraction of the rabbit thoracic aorta and mesenteric arteries were compared to papaverine [14]. Cinnarizine was found to block the Ca-evoked contraction of the depolarized vessels but was less effective against the noradrenaline-induced contraction of the mesenteric arteries and even failed to antagonize the response of the thoracic aorta to noradrenaline. Cinnarizine was effective in mesenteric arteries exposed to noradrenaline by blocking the Ca_0_ dependent response, while papaverine was less effective towards the Ca_0_ dependent response but efficiently inhibited the initial fast response more. Cinnarizine was less effective or even failed to inhibit the response produced by thoracic aorta to nonadrenaline. These results suggested reduction of membrane permeability to extracellular calcium was the mechanism by which cinnarizine acted. The report also suggested that papaverine inhibits the contraction to less extent than that of cinnarizine. It attenuates vasoconstriction action of many endogenous substances by modulation of calcium fluxes. Cinnarizine is known to promote the cerebral blood flow. So have wide application in treatment of cerebral apoplexy, cerebral arteriosclerosis, and posttraumatic cerebral symptoms [15]. According to a recent paper, the effect against vestibular vertigo was due to inhibiting potassium (K^+^) currents, activated by heightened hydrostatic pressure on the hair cells, instead of blocking calcium channels [16]. It inhibits contraction of smooth muscle cells by blocking L-type calcium channels [5]. Reports on inhibitory action on 5 H serotonin uptake by platelets are also available [17]. The drug inhibits 5-HT more efficiently than K^+^-induced contractions [18]. Its moderate antagonistic properties at dopamine D2 receptors are known to resemble the mechanism of action of most antipsychotic drugs. The active metabolite of cinnarizine, [1-(diphenylmethyl)-4-[3-(4′-hydroxyphenyl)-2-propenyl] piperazine, has higher affinity for D2-receptors than the parent drug that indicates significant role in inducing parkinsonism as adverse effect on chronic medication [19]. Recently cinnarizine has been reported as melanogenesis inhibitors. Additionally the drug was suggested to be used in cosmeceuticals as skin whitening agents for treatment of skin hyperpigmentation [20]. The pharmacodynamic features are tabulated in Table 1.

## 2. Clinical Therapeutics and Trials

Clinically, cinnarizine is used for treatment of vertigo and prevention of motion sickness, due to its calcium channel blocking activity. Vertigo indicates a sensation of false movement (generally described like rotation) and vestibular vertigo may be induced by increase in the endolymphatic pressure that activates pressure-dependent K^+^ currents (*I*_K,*p*_) in vestibular hair cells; this modulates transmitter release. Cinnarizine acts by enhancing the transmitter release in vestibular type II hair cells by inhibition of (*I*_K,*p*_), thus enhancing depolarization and increasing the voltage-dependent activation of Ca^2+^ currents [16]. The therapeutics of cinnarizine has been proved employing clinical trials and the reports are compiled in Table 2. Briefing the reports, the early trials were conducted for vertigo, chronic asthma. Additionally, cinnarizine has evoked clinical interest for other disorders. Consequently, recently summarized reports suggest clinical application of drug for migraine prophylaxis. On the other hand, cinnarizine is known to aggravate Parkinson's disease [21]. When used in average dose of 150 mg daily, it can aggravate motor function in patients with Parkinson's disease; however, the effect was reversible and disappeared upon withdrawal of drug. Its use with caution is recommended.

## 3. Commercial Products

**Figure 2:**
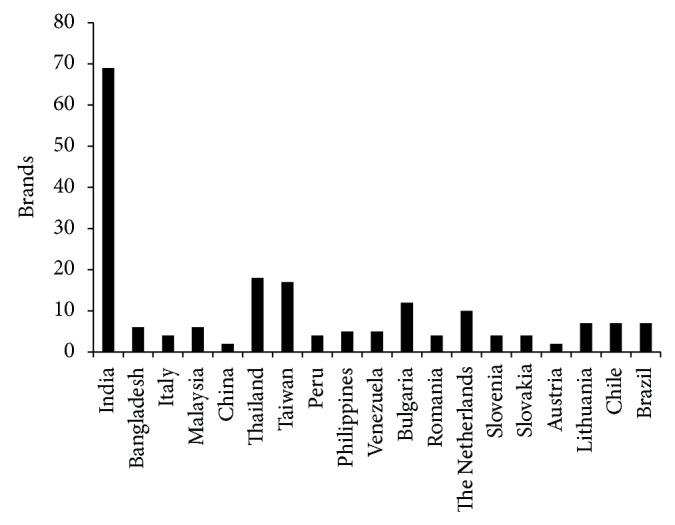
Global distribution of cinnarizine commercial brands.

Commercial cinnarizine is available as tablets and capsules usually administered 2-3 times a day. Drowsiness to deep sleep and dizziness are some common adverse effects. High dose should be used with caution in patients with hypotension, owing to the possibility of further decrease in blood pressure, and is contraindicated in pregnancy due to teratogenic and embryocidal effects on the foetus. The drug can only be prescribed if the potential benefit justifies the potential risk to the foetus. Cinnarizine is widely marketed as combination medication with other drugs. In the Indian domestic pharmaceutical market, its combination with domperidone is predominant, whereas combination with other drugs such as dimenhydrinate, dihydroergocristine, chlorcyclizine, phenylpropanolamine, and piracetam are available with overseas pharmaceutical companies. Some of the combination brands are enlisted in Table 3. Cinnarizine pharmaceutical products are commercialized all over the world (Figure 2). India leads in terms of commercialization of different brands of cinnarizine comprising approximately more than 50 brands including both drug in alone as well as in combination with other drugs. Among all brands Stugeron has the highest distribution in around 39 countries [22, 23].

## 4. Bioavailability Enhancement

It has been accounted in research that bioavailability of cinnarizine is remarkably influenced by gastric acidity [24], displaying its pH dependent solubility and in turn dissolution. The marketed dosage forms account for low and erratic bioavailability [25], mainly due to the above-mentioned pharmacokinetic reasons. Also, high dosing frequency is needed because of its short elimination half-life [5]. Its poor solubility at high pH results in incomplete absorption. Being a weak base it is very soluble in acidic solutions and clinical absorption of cinnarizine was found to depend on the gastric acidity of patients. Cinnarizine showed rapid dissolution in individuals having high gastric acid content (pH = 1.2), providing good absorption characteristics, whereas, in case of low-gastric acid content (pH = 6), reduction was observed in *C*_max⁡_ and AUC approximately 75 to 85% [26].

Literature retrieval on enhancement in solubility and bioavailability of cinnarizine highlighted various effective approaches since 1984 till date. In 1984, Tokumura et al., [27] in his report, claimed 30 times improved dissolution for inclusion complex of cinnarizine with *β*-cyclodextrin than the drug alone, whereas no difference in bioavailability was observed. Further working with the cinnarizine *β*-cyclodextrin complex, Tokumura with other research devised a method for improvement of bioavailability on simultaneous administration of proposed *β*-cyclodextrin with competing agent. It was found that the penetration rate constant of cinnarizine in complex decreased which was restored on addition of competing agents. These agents compete with *β*-cyclodextrin in complex formation, thus keeping more available free drug to penetrate the lipid barrier. In the same content, two papers were projected. One was based on the investigation of the competing ability of DL-phenylalanine to improve the bioavailability of cinnarizine [28]. On simultaneous administration of DL-phenylalanine with cinnarizine *β*-cyclodextrin complex, bioavailability was remarkably enhanced. This enhancement depends on the dose of competing agent administered. It was concluded that a minimum effective dose of 1 g of DL-phenylalanine might be required for every 50 mg of cinnarizine in *β*-cyclodextrin complex. On the similar lines, the ability of L-Leucine and L-Isoleucine was also investigated for competing effect in gastrointestinal tract [29]. As a result the *C*_max⁡_ was increased to 2.7 and 1.9 times as compared to that of drug alone and its *β*-cyclodextrin, respectively. Conclusively, L-isoleucine showed stronger competing effect than L-leucine coupled with significantly improved bioavailability of the drug. In the next year, in 1987, enhancement of bioavailability of cinnarizine as its oleic acid solution in hard capsule for oral administration was reported [30]. Use of lipid vehicle for oral administration of drug was ascertained to improve the bioavailability when compared to cinnarizine tablet. When investigated in beagle dogs, the *C*_max⁡_ and AUC for oleic acid solution of cinnarizine were remarkably improved by 2.9 and 4 times than that of tablet, respectively. The results were encouraging and somewhat similar to those obtained with simultaneous administration of *β*-cyclodextrin and competing agent discussed above. In yet another report the *β*-cyclodextrin derivatives, SBE4-beta-CD and 2-hydroxypropyl-*β*-cyclodextrin (HP-beta-CD), were used to form complex with cinnarizine to enhance the oral bioavailability [31]. The absolute bioavailability of solution or capsule containing drug alone was compared to *β*-cyclodextrin derivatives complex of drug as a solution and in capsule. To assess the bioavailability, cinnarizine was administered to beagle dogs in the dose of (and an amount equivalent in case of *β*-cyclodextrin derivatives complex) 25 mg orally and 12.5 mg/mL intravenously. The bioavailability of cinnarizine was 8 ± 4% for solution of drug and 0.8 ± 0.4% for capsule filled with drug alone. This was observed to be significantly increased to 55–60% and 38 ± 12% in case of *β*-cyclodextrin derivative complex of drug formulated as suspension and that filled in capsules, respectively. The higher bioavailability achieved in case of cinnarizine *β*-cyclodextrin derivative complex formulated as suspension than that filled in capsules was attributed to rapid dissociation of drug from its inclusion complex, thus causing improved absorption.

The enhancement in bioavailability was also attributed to enhancement in dissolution. As mentioned earlier the drug exhibits dissolution rate limited absorption. Based on this concept, a simple technique of preparing solid dispersion of drug by fusion method was explored. The study was aimed at achieving increased dissolution and sustained release effect separately. The purpose was served by the use of two lipid carriers separately, Gelucire 44/14 (gastric acid soluble) and other Compritol 888ATO (gastric acid insoluble). In case of Gelucire 44/14, the results suggested initial rapid release of drug which then sustained for over 5 h giving a release of approximately 90%, whereas Compritol 888ATO at a maximum ratio of 1 : 1 (drug: compritol888ATO) more prolonged the release when compared to pure drug and commercial tablet supported the efficiency of solid dispersion to enhance the bioavailability [32].

The drug being a weak base exhibits higher solubility in acidic environment of stomach and hence dissolution. On the other hand it displays poor dissolution in basic environment of small intestine. The inhibited drug release of drug in basic environment can be raised by incorporation of acidifiers in the formulation that create acidic environment. Incorporation of acidifiers like citric acid or fumaric acid in matrix tablet to raise the solubility and hence pH independent drug release was also suggested [33]. The drug release under the influence of acidifiers was examined by preparing three combinations, formulation containing only citric acid (70 mg) or fumaric acid (70 mg) separately and in combination with citric acid and fumaric acid (35 : 35 mg) too. Drug dissolution study was carried out using modified flow through cell. The acidifiers are able to reduce the pH of the intestinal and maintaining it for extended period of time, thus providing sustained drug release. The drug release was 57.3% and 53.5% after 8 h which increases to 75.2% and 66.2% after 12 h for citric acid and fumaric acid, respectively, whereas it was only 26.5% after 12 h. This shows better efficiency of citric acid for maintaining low pH environment. The lack point is depletion of acidifier from the tablet matrix, the rate of which is dependent on type of acidifier. Citric acid was quickly depleted in comparison to fumaric acid. Acidifiers are also known to resist precipitation of drug at higher pH. Hence, the result suggested that proper selection of acidifiers may give pH independent release profiles that in turn would lead to higher bioavailability.

Conclusively, the problematic issue of low and erratic bioavailability of poorly aqueous soluble drug, cinnarizine, was a topic of interest to researchers. The efforts were dedicated from the use of well known *β*-cyclodextrin complex to some recent use of liquid cubic phases and acidifiers like citric acid and fumaric acid. Additional use of competing agents (such as DL-phenylalanine and L-isoleucine) in combination with *β*-cyclodextrin complex and lipid vehicles like oleic acid was proved to be advantageous and further increased the bioavailability of drug. On establishment of comparison between these mentioned techniques to that of cinnarizine in alone or its marketed preparations, the results were encouraging with remarkable enhancement in bioavailability of cinnarizine.

## 5. Drug Delivery Systems

Various dosage forms/delivery systems have been approached to solve the solubility and bioavailability issue. These include fast dissolving tablets, nanoemulsions, microparticles, and as gastroretentive systems. To the best of our knowledge, here is the compilation of research reports, till date, on delivery related approaches to overcome the pharmacokinetic constraints of cinnarizine. These systems have their own advantages and limitations as listed in Table 4. Critical analysis based on in vitro results revealed improved bioavailability of cinnarizine formulated as various dosage forms, when compared to marketed preparation. In contrast to in vitro results, the in vivo data for improvement in bioavailability is limited and efforts are needed to be made to assess the bioavailability and performance of cinnarizine in vivo.

### 5.1. Fast Dissolving Tablets

Cinnarizine could serve as suitable candidate for fast dissolving tablet that may improve the bioavailability and patient compliance. Fast release tablet of cinnarizine was prepared [34]. The in vitro dissolution rate of granules was increased (80–90%, 60 min) on comparison with drug alone (less than 50%, 80 min), because of the higher hydrophilic character imparted by hydrophilic carriers and reduction in drug crystallinity in case of granules. The report suggested the use of fluidized hot melt granulation method as an advantageous means of producing granules of cinnarizine with PEG 6000 as a melt binder, which does not need solvents or water. Chitosan based orodispersible tablet of cinnarizine was reported with the aim of improving absorption of drug which steps towards improved oral bioavailability [35]. The study guided the use of chitosan based orodispersible tablet to be a valuable alternative in respect to use of expensive superdisintegrant. The prepared tablets have advantage of disintegrating within few seconds without need of water, which lead to enhanced absorption of drug. The disintegration time of optimized formulation was 11 sec giving 90% of drug release in a time period of 5 min. According to this report, the researchers assumed that if 90% of drug is released in the dissolution media in vitro, then it will exert full effect in vivo. So based on the results, the sufficient dissolution was assumed to enhance the absorption of drug that leads to improvise the bioavailability when assessed in vivo providing effective therapy. This ultimately aims towards improving patient compliance with main focus on children and elderly patients. Basu et al. [36] developed fast dissolving tablet of cinnarizine using combination of superdisintegrants sodium starch glycolate (SSG) and croscarmellose sodium (CCS) and camphor as a subliming material. Camphor was added to aid the porosity of the tablet. The optimized formulation (SSG = 12 mg, CCS = 12 mg, and camphor = 10 mg) when compared to widely used commercial tablet (Stugeron) was reported to be more effective with drug release of 99% in 16 min. In another report, fast dissolving tablets of cinnarizine were prepared by sublimation technique and regarded as effective alternative approach compared to use of expensive excipients [37]. The tablets were evaluated for various precompressional and postcompressional parameters. The precompressional evaluation revealed good free flowing properties within the prescribed limit. Considering the pharmacopoeial test, the drug was uniformly distributed throughout the tablet with high drug content (≥101.5%). The average percent deviation was less than ±7.5% that provided good uniformity. Apart from these, the friability was < 1 in all formulations. The subliming agents have directly proportional effect on the disintegration of tablets which further affect the drug release. The optimized formulation was reported to have *t*_50%_ of 6.25 min and *t*_90%_ of 19.74 min. It was stated that upon storage the disintegration of tablet was significantly decreased (*P* < 0.05). The report concluded the effectiveness of sublimation method as an alternative approach for fast dissolving tablet of cinnarizine to reach the optimum point in shortest time with minimum efforts. In yet another report, oral dispersible tablets were prepared by three different methods: effervescent, superdisintegrant addition, and sublimation method [38]. The tablets showed total release of drug in a period of 6 h with total disintegration in 25.3 sec. The report concluded that the superdisintegrant addition method with L-hydroxypropyl cellulose (10% w/v) was best to fulfill the aim. Recently Heer et al. [39] developed fast dissolving orodispersible film and fast dissolving tablet of cinnarizine and assessed the effect of superdisintegrants. For this purpose, three different superdisintegrants, sodium starch glycolate, crospovidone, and croscarmellose sodium, were used. The tablet was prepared by direct compression method and solvent casting method was employed for preparation of orodispersible film. The two preparations were evaluated for weight variation, thickness, drug content, folding endurance, and tensile strength (for orodispersible film). As a result, among three superdisintegrants, crospovidone at 4% w/w was proved to have promising superdisintegrant property required to prepare fast dissolving formulations. Fast dissolving orodispersible film was concluded to be better over fast dissolving tablet in terms of faster drug release with better flexibility suggestive to patient compliant dosage form.

### 5.2. Lipid Based Drug Delivery Systems

Lipid based drug delivery systems were topic of interest to scientist as novel drug delivery platform [40] and well known to improve the oral bioavailability of poorly water soluble drug that belongs to BCS class II and IV. The most interesting mechanism of enhanced bioavailability was regarded as improved absorption due to enhancement of gastrointestinal solubilisation [38]. The factors that relate to bioavailability from lipid formulations include digestion of lipid, the mean emulsion droplet diameter, the lipophilicity of drugs, and types of lipids. The lipids are preferred for oral administration as they can be formulated in solution, suspension emulsions, self-emulsifying, and microemulsions [41]. Lee et al. [42] reported lipid based formulation of drug and studied the effect of lipid on drug absorption. The in vivo absorption study revealed higher bioavailability with long chain triglyceride than medium chained triglyceride, giving a value of 32.1 ± 2.3% and 16.6 ± 2.3%, respectively, but was independent of quantity of lipid administered. However, the report concluded that the bioavailability was observed to increase with lipid dose of 125–250 mg but reached static point at 500 mg. Improvement in solubility of drug in self-nanoemulsified drug delivery system was reported [43, 44]. Lipids have the capability to keep drug in solution upon dispersion in aqueous medium. SNEDDS containing combination of Brij 97 (17.6% w/w) and PEG 400 (8.8% w/w) revealed good bioavailability in vivo with an area under curve (AUC_0–48 h_) of 773 ± 148 ng mL^−1^ h^−1^. A double peak phenomenon was observed for plasma profile. The reason for high bioavailability was due to presence of high content of surfactant. This helped in keeping cinnarizine solubilized even at lower part of gastrointestinal tract; thus it is available for absorption for longer duration. It was observed that the biorelevant media greatly affected the type and size of droplets formed. Upon dispersion in media containing low bile acid content, vesicles were formed, whereas it was micelle formation in presence of higher bile acid content. The resulted nanostructured composition influenced the solubilization capacity of composition which potentially affected the absorption of drug from resultant media. This research report on SNEDDS suggested the approach to be suitable for better absorption of drug. Again working with SNEDDS, the researcher published another paper this time to assess the effect of increasing loading level of cinnarizine. Three formulations were developed, SNEDDS_High_, SNEDDS_Medium_, and SNEDDS_Low_ with 50, 25, and 12.5 mg of drug, respectively. The in vitro lipolysis at pH 6.5 showed that SNEDDS with high concentration of cinnarizine leads to precipitation of drug, whereas it decreased in drug loading. There was no significant difference in AUC, *T*_max⁡_, and *C*_max⁡_ for the three formulations. The *T*_max⁡_ was 1.8 ± 0.14 h, 23 ± 0.64 h, and 3 ± 0.71 h after 1 g SNEDDS_High_, 2 g SNEDDS_Medium_, and 4 g SNEDDS_Low_, respectively. The AUC obtained was 355 ± 74, 485 ± 68, and 392 ± 52 ng/mL∗h^−1^ for the three formulations in order of their increased drug loading, respectively. The report presented that, on oral administration, the drug loading level in SNEDDS and amount of Snedds vehicle did not affect the bioavailability [45]. A lipid based liquid crystalline matrix of cinnarizine was prepared for sustained release delivery of drug which ultimately enhances the bioavailability [46]. The lipids used were glyceryl monooleate and oleyl glycerate. In vitro lipolysis study suggested less susceptibility of oleyl glycerate to hydrolysis by pancreatic lipase than that of glyceryl monooleate. In order to investigate the improvement in bioavailability, cinnarizine was orally administered in the form of suspension or solution in glyceryl monooleate or olely glycerate. The bioavailability was more enhanced by administering cinnarizine with oleyl glycerate and appeared to be 344% (aqueous suspension formulation assigned a reference bioavailability of 100%). The report concluded the efficiency of lipids that have an ability to form liquid crystalline structures in excess water, to be set forth as an application for oral sustained delivery of drug to enhance bioavailability. Vithlani et al. [47] developed SEDDS with the aim of improving aqueous solubility and dissolution profile. The SEDDS with 30% w/w of Capmul PG-12 when evaluated in phosphate buffer, pH 7.2, provided remarkable enhancement in drug solubility in phosphate buffer(s) with rapid drug release. The SEDDS showed least solubility of drug to be 1500 *μ*M in phosphate buffer, pH 7.2. It was concluded that the SEDDS prepared by combination of Capmul PG-12, Tween 20, and Cremophor RH 40 can be a suitable alternative dosage form to fulfill the aim of the research that is to improve solubility and dissolution of drug. SNEDDS of the drug was also reported with the aim of exploring the effect of type of lipid on solubility and drug release [25]. Proper balance between rapid and efficient self-emulsifying ability, higher solubility of drug, maintaining high amount of drug in solution after aqueous dispersion (at least 85%), and lower droplet size upon aqueous dilution were the selection criteria for optimal formulations. The length of lipid chain significantly influenced the solubility of drug and long chain lipids showed higher solubility than medium chained. In terms of glyceride composition the medium chain monoglycerides had better solubility than medium chain triglycerides. Furthermore, the unsaturated fatty acids with long chain lipids resulted in higher solubility than the above two. SNEDDS showed significantly better release in vitro in simulated gastric fluid (SGF) (to mimic the in vivo condition more accurately) than marketed preparation, Stugeron. The SNEDDS showed a release of 84–95% after 15 min which was only 66% in case of Stugeron in simulated gastric fluid. After 15 min of shifting into simulated intestinal fluid, the marketed preparation showed comparatively much higher drug precipitation (83%) than the SNEDDS which causes only 7–23% of drug precipitation. This suggested superiority of SNEDDS to resist drug precipitation on shifting to basic pH. A paper was also reported to enhance the oral bioavailability of cinnarizine from its medium chain lipid solution formulation. Lipid based formulation of cinnarizine was administered intraduodenally and perorally to rats in order to assess the contribution of dispersion and digestion in the stomach towards bioavailability. The bioavailability was observed to be decreased after intraduodenal administration. The difference in bioavailability was also observed for medium chain and long chain triacylglycerides owing to difference in the dispersion and digestion in the gastric and intestinal compartments. The difference between the bioavailability was large in case of medium chain lipid when compared to long chain lipid in case of both peroral and intraduodenal administration. The result suggested that the passage of medium chain lipid formulation through stomach led to critical gastric digestion than long chain lipid formulations. The report concluded that rapid transfer of medium chain formulation to intestinal mixed micelles possibly was achieved by efficient dispersion and partial digestion in stomach. Also absorption in the upper intestine prior to drug precipitation was preferred [48].

In yet another report the high solubility and stability of cinnarizine loaded emulsion (CLE) had been proved [15]. Egg lecithin was used with the aim of minimizing drug degradation from both water and oil. The drug was localized in the interfacial lecithin layer of emulsion so as not to contact with oil or water medium. On further working with the CLE, in next year the researchers provided reports for pharmacokinetics and tissue distribution of drug [49]. When assessed in vivo in rats, the novel formulation (CLE) revealed significantly higher AUC and lower clearance and distribution volume. On i.v. administration of 2 mg/kg of cinnarizine loaded emulsion, the AUC was observed to be 1879.556 *μ*g/Lh^−1^, whereas it was 865.725 *μ*g/Lh^−1^ for aqueous cinnarizine solution prepared using Tween 80 (1 g) and 1,2 propylene glycol (10 g). The result revealed lower clearance (0.947 L/h/kg) for drug loaded emulsion. Consequently, circulation in blood stream achieved for longer duration resulted in better therapeutic effects. The tissue distribution exhibited lower side effects due to drug precipitation in vivo. The system provided better bioavailability, improving the pharmacokinetics of drug. Contributing to bioavailability of drug, researchers explored self-microemulsifying system (SMEDDS) [50]. SMEDDS is known to spread readily in the gastrointestinal tract, and the agitation provided by digestive motility of stomach and the intestine results in self-emulsification. SMEDDS containing oleic acid (16.66% w/w), Tween 80 (55.55% w/w), and Transcutol P (27.77% w/w) improved the solubility and drug release when compared to marketed formulation of cinnarizine. In the report, the authors have explained formation of small sized particles, leading to rapid drug release of 81.96% in 5 min which slightly increases to 87.67% in 60 min. This suggested the potential use of SMEDDS to improve oral bioavailability by overcoming the solubility issue. In addition to this, SMEDDS of cinnarizine was prepared to characterize a better understanding of the mechanisms behind the precipitation of cinnarizine during in vitro lipolysis of a self-microemulsifying drug delivery system [51]. In the period of in vitro lipolysis of the SMEDDS with or without cinnarizine, samples were withdrawn at different time points and ultracentrifuged. Initially cinnarizine content in pellet was 4% which increased to 59% during lipolysis. There was good correlation between precipitation of cinnarizine and degree of lipid digestion. Dissolution was performed in biorelevant media and initially the dissolution rate of cinnarizine from pellets containing precipitated cinnarizine was 10-fold higher than dissolution from blank pellet spiked with crystalline cinnarizine, which increased to more than 50% drug dissolved in the first minute. Pellets were further characterized by X-ray powder diffraction (XRPD) and polarized light microscopy (PLM). Results indicated that either amorphous form or a molecular dispersion of cinnarizine resulted in precipitation during in vitro lipolysis of SMEDDS, thus improving dissolution profile. Recently SNEDDS was also investigated [52] to eliminate the effect of food on cinnarizine absorption. A nutritional drink, Fresubin energy (200 mL), was used to simulate the fed state conditions to induce food effect. The SNEDDS capsules or tablets in fed state were assessed for bioavailability by administering SNEDDS equivalent to 50 mg of drug orally to male beagle dog (10–13.5 kg) with one week wash-out period between dosing. The dogs were fasted overnight for about 20–24 h. The nutritional drink was administered 30 min prior to dosing. Pentagastrin (6 *μ*g/kg) was administered to dogs in order to control the gastric pH. The result also established the effect of food to extend the *T*_max⁡_ to 2.5 times than fasted state and increased bioavailability for conventional tablets (*F*_fasted_ = 20.9 ± 5.7 and *F*_fed_ = 53.8 ± 30.1). In contrast to this the food condition did not affect the absorption of drug from SNEDDS capsules and tablets, while *T*_max⁡_ was observed to extend the absorption and thus enhanced the bioavailability. The fed state showed 2.5 and 3.3 times longer *T*_max⁡_ than fasted state for SNEDDS tablets and SNEDDS capsules, respectively. The bioavailability of SNEDDS capsules was higher at both fed (79.3 ± 14.7) and fasted state (58.1 ± 16.7) than that of SNEDDS tablet (*F*_fed_ = 43.7 ± 6.7 and *F*_fasted_ = 32.7 ± 11.5). SNEDDS formulation was observed to diminish the variation in absorption at fasted and fed conditions, leading to overall increase in bioavailability compared to conventional tablets. At the end, the conclusive points were drawn that food affects the absorption of BCS class II drug causing increased bioavailability in fed state which can be significantly reduced by the use of SNEDDS capsules and tablets. In the same context, another report was published in the same year to predict the performance of two above discussed lipid formulations (SNEDDS capsules and SNEDDS tablets). The two formulations were compared with conventional tablets on the basis of gastrointestinal in vitro lipolysis model in both fasted (pH = 2) and fed conditions (pH = 6). The in vitro lipolysis model was supposed to simulate the digestion in the stomach and intestine. To mimic the lipolysis events in fed state conditions, fat milk (3.5%) was administered with 0.4% mg/mL of pepsin. At gastric step during fasted state, a high concentration of drug was found in aqueous phase, whereas it was lower for SNEDDS tablet which can be explained on the basis of presence of alkaline excipients that lead to increase of the pH to 3.64 and cause lower solubility. The solubilization of drug remained constant for SNEDDS formulations upon initiation of duodenal step suggested the role of lipid to enhance the solubilization of cinnarizine. The total amount of cinnarizine recovered from fasted state lipolysis was almost similar for all formulation with an average of 84 ± 12%. On examining fed state condition, approximately 1% of cinnarizine from SNEDDS was shown in aqueous phase in gastric step, whereas it was only 0.1% for conventional tablet. On passing into duodenal step, SNEDDS formulations showed an aqueous cinnarizine content of 40% after 83 min and maintained it throughout the lipolysis, while the conventional tablet lost solubilization capability decreasing to 25.4 ± 1.7% at the end. When compared to in vivo data provided by paper discussed above, these in vitro results were in accordance with in vivo. The order was similar as SNEDDS capsules > SNEDDS tablet > conventional tablet for performance in vitro and in vivo. More conclusively, the in vitro fasted state model was able to predict the correlation with in vivo study, whereas the fed state model cannot exactly establish a correlation but was able to predict the best formulation similar to that predicted by in vivo study [53]. In the path of lipid based nanosized delivery of cinnarizine, a report was published to stabilize the nanosuspensions by the utilization of bead layering. The nanosuspension of drug was coated on sugar beads by the use of fluidized bed pellet coater with Wurster insert. These nanosuspensions showed complete dissolution in 15 min, whereas it was only 11% in 1 h for unmilled powder. The coated nanocrystals resulted in slower release of cinnarizine than that of original nanosuspensions due to formation of reagglomeration upon release from coating. The conclusive point suggested influence of drug physicochemical properties on in vitro dissolution after bead layering [54].

Utilization of lipids for pharmaceutical and biomedical applications was known to overcome the issues related to biocompatibility and biodegradability. In this respect the unique mesophasic structure of lipids such as cubic, hexagonal, and sponge phase structures provides advantage of stability and production feasibility. The cubic structure has advantage of improved stability, bioadhesivity, and biocompatibility, whereas the hexagonal arrangement exhibited hexasomes accounts for encapsulation of different drugs with high stability [55]. The similar reports on cinnarizine are discussed further. The contribution towards improved absorption of cinnarizine was made by novel approach of cubic liquid crystalline phase [56]. The cubic phase improved the solubility of cinnarizine and provided sustained release of drug. In addition to this, another highlight of cubic phase is its temperature sensitivity in the range of 20–37°C suggesting it to be a putative material for the purpose of in situ gelation. The in vivo absorption study was performed on anaesthetized male Sprague-Dawley rats (280–320 g). Cubic phase equivalent to 5 mg of cinnarizine was administered intraduodenally. The result illustrated large difference in the rate of cinnarizine absorption from cubic phase and suspension. The absorption profile was more sustained and continued beyond 8 h after dose period for cubic phase, thus supporting its application to improve bioavailability. The *C*_max⁡_ obtained for cubic phase was 26.2 ng/mL after 4.7 h, whereas it was 99.9 ng/mL after 2.2 h for suspension proving sustained absorption for former case. Another remarkable contribution towards improved bioavailability was made recently [57]. The report was the first to reveal the ability of liquid crystalline nanostructured particles (cubosomes) to attain sustained absorption of cinnarizine. These cubosomes were developed using phytantriol (an alcohol) and glyceryl monooleate (a lipid). Phytantriol cubosomes showed improved results with sustained absorption beyond 48 h. The oral bioavailability was improved to 21% compared to suspension (*F* = 9%) and oleic acid emulsion (*F* = 12%). In such cases of nondigestible phytantriol cubosomes, the slow release can be facilitated by stomach that acts as a nonsink reservoir. Thus, the report suggested the potential use of nondigestible liquid crystalline nanostructured particles as a novel approach for sustained oral drug delivery systems leading to improvise the bioavailability of poorly water soluble drugs. In the same year the researchers made another contribution towards nanosized lipid formulation [58]. Hexagonal liquid crystals (hexosomes) of cinnarizine were prepared with the aim of sustaining the absorption for increment in bioavailability. The pharmacokinetics was evaluated on oral administration of bolus lipid solution of drug in selachyl alcohol to rats. The results revealed that, after oral administration, sustained plasma profile with 20–40 ng/mL of drug maintained over the first 24 h was observed. When compared to suspension, the *t*_max⁡_ was longer for hexosomes (1 h and 23.5 ± 5.9 h resp.). The absolute oral bioavailability was improved for hexasomes (17%) compared to that of suspension (9%). Selachyl alcohol is not susceptible to digestion which attributed to prolong the absorption as similar to nondigestible phytantriol cubic system discussed above. The nanostructure of hexasomes remained unaffected by simulated intestinal fluid and drug. The report concluded the potential use of nondigestible liquid crystalline systems with additional advantages of nanostructured particles as novel system for sustained drug delivery.

### 5.3. Gastroretentive Systems

The gastroretentive preparation of cinnarizine was first reported in 1989 [59]. The research reported two preparations: one was a buoyant tablet containing powdered soyabean, drug, and sodium bicarbonate and the other was laminated floating film comprising of a drug film, an effervescing film of sodium bicarbonate, and an outer drug release regulating film. The in vitro studies assured the suitable floating ability and sustained release property of the two formulations. Linear dissolution was observed after 2 h in case of laminated film not similar to tablet due to structural difference. The absorption study was done by oral administration of buoyant tablet and film in beagle dogs. As a result, cinnarizine was observed in blood even 24 h after oral administration, concluding the efficiency of the two preparations as sustained delivery systems so as to upgrade the absorption and bioavailability of cinnarizine. Floating microballoons were prepared by diffusion solvent evaporation technique using 2^3^ factorial design to improve bioavailability [60]. The preparation was supposed to provide sustained delivery of cinnarizine. In vitro drug release was performed in pH 1.2 for 8 h and then in pH 7.4 for 2 h. Microballoon released approximately whole drug during 10 h period with sufficient buoyancy. The microballoons prepared with combination of Eudragit S100 and Eudragit RL in a ratio of 1 : 3 proved most effective results, suggesting the efficient use of microballoons for oral sustained delivery of cinnarizine. Nagarwal et al. [61] developed single unit matrix tablet dosage form for gastroretentive purpose. These tablets were prepared using four viscosity grades of HPMC with gas forming agent. The sustained drug release was achieved due to rapid hydration of polymer on the surface of floating tablet, thus forming a gel layer surrounding the matrix. This controls penetration of water to the center of matrix tablet and retards the release. It was reported that, with increase in viscosity of polymer, drug release rate was increased. While studying the release rate with different polymers, it was also revealed that the drug release rate depends on the surface area as well as hydration capacity of polymer. Based on these results, the optimized formulation containing HPMC K100LV (90 mg) was compared (in vivo) to oral suspension and bioavailability was studied. The absorption phase was slow and prolonged in case of matrix tablet, thus indicating prolonged plasma concentration. The overall bioavailability of floating matrix tablet was 2.85 times greater than that of oral suspension. In 2012, floating tablet of cinnarizine in combination of domperidone maleate was developed for gastroretentive delivery. The pH independent polymer, Methocel polymer of different viscosity grades, was used at concentration of 20–50% w/w to prepare floating tablets. The formulation was reported to remain buoyant for 12 h with lag time of 10–15 sec in 0.1 N HCl. Sodium bicarbonate (15 mg) and sodium alginate (18 mg) were used as gas generating agent necessary to impart sufficient buoyancy. So the report suggested improved retention of tablet of combination of two antiemetic drugs at gastric pH that was supposed to have improved therapeutic effects [62].

Multiparticulate systems reported as gastroretentive floating microspheres were developed to assess the effect of formulation variables on microsphere formation [63]. Microspheres were developed using Acrycoat S100, Eudragit RS100, and ethyl cellulose as gastroretentive polymers. When assessed in vitro in 0.1 N HCl, the microspheres remained buoyant for 12 h. Among these, microspheres containing Acrycoat S100 (100 mg) showed best results for sustained drug release (98.14%), floatability (72%) in 12 h, and stability. Another effort to sustain the release of cinnarizine was attained by preparation of drug loaded chitosan methylcellulose interpenetrating polymer network (CS/MC IPN) microspheres intended for mucoadhesive gastroretentive application. The study explores the interpenetrating network of two biopolymers, chitosan and methylcellulose, to prolong the residence of microspheres in the stomach. The microspheres were prepared using central composite design at three different levels of polymeric ratio and glutaraldehyde. The formulations were evaluated for degree of mucoadhesiveness which was correlated to amount of polymer and interpenetrating network of polymers. For optimized formulation the percent of mucoadhesion was 73.85 ± 2.78 at 4 h with *t*_50_ of 432.21 ± 26.15 min. The other observed results were for mean diameter (61.32 ± 1.38 *μ*m), swelling index (2.38 ± 0.06), and entrapment efficiency (84.13 ± 1.32%). The report suggested the utilization of chitosan methylcellulose interpenetrating polymer network (CS/MC IPN) as potential approach to impart mucoadhesive property for gastroretentive delivery of weakly basic drugs [64]. Recently in the past year, Patel et al. [65] prepared mucoadhesive microparticles by supercritical fluid method using chitosan as mucoadhesive polymer. The efficiency of this physical coating process for producing microparticles was established by X-ray powder diffractometry and differential scanning calorimetry. The prepared microparticles were proposed for better bioavailability than the suspension in test animal due to gastroretention. The comparison was done in vivo by administering both suspension and mucoadhesive microparticles to rabbit in fasted state. The mucoadhesive microparticles showed sustained drug release for more than 20 h. Good mucoadhesive properties and strong adherence of microparticles to gastric mucus layer were established both in vitro and in vivo, resulting in gastroretention for an extended period of time. These formulations accounts for sustained effect of drug reducing the dosing frequencies. In the same year mucoadhesive tablets of cinnarizine were developed using Eudragit RLPO as polymer with the aim of investigating the effect of iron oxide in the formulation [66]. The mucoadhesive tablets were prepared at different concentrations of Eudragit RLPO, iron oxide, and PVP K 30 and evaluated for mucoadhesive strength, *t*_50%_, *t*_90%_, and mean dissolution time effect of iron oxide was investigated. Ex vivo mucoadhesion test was performed employing texture analysis. The result clarifies the synergistic effect of combination of Eudragit RLPO, iron oxide, and PVP K 30 to enhance the mucoadhesive strength when compared to their individual effect. As per the results the mucoadhesive tablets containing 8.58% w/w Eudragit RLPO, 6.68% w/w iron oxide, and 7.86% w/w PVP K 30 were selected to be optimized and fulfilled maximum requirements with better mean dissolution time (266.44 ± 3.82 min), *t*_50%_ (124.94 ± 5.40 min), *t*_90%_ (755.53 ± 4.29 min), and desirability of 0.727. The Incorporation of iron oxide was found to enhance the mucoadhesive strength due to induction of ionic interaction. Retardation of drug release with mean dissolution time of 229.56 ± 3.30 min at higher concentration of iron oxide was achieved due to reduction in polymeric chain disentanglement and polymer erosion caused by the entrapment of iron oxide in the polymeric chain network.

A novel approach of gastroretention with controlled release was recently negotiated by preparing an unfolding type film of cinnarizine [67]. The polymeric film was procured by solvent casting method using ethyl cellulose and hydroxyl propyl methyl cellulose K15 and folded in hard gelatin capsule. The presence of stearic acid in polymeric film has crucial role. As obtained by in vitro drug release study, the film describes fast release of drug in the first hour due to initial leaching out of drug, which was followed by slow release by diffusion through hydroxyl propyl methyl cellulose and ethyl cellulose providing more steady controlled release giving total release of 81.93% in 13 h. The result also suggested more appropriate use of unfolding film in simulated gastric fluid when compared to roll folded pattern. The research strongly indicated the gastroretentive potential of prepared dosage form which can be explored further for in vivo studies.

Apart from these preparations, enteric microparticles were developed [68]. Cinnarizine is known to precipitate at high pH on gastric emptying and show slow and erratic bioavailability. The microparticles with enteric coating were prepared to circumvent this obstacle. Microparticles prepared using pH responsive polymer Eudragit L by emulsion solvent evaporation method showed improved bioavailability. The microparticles inhibited the in vitro release of drug under gastric conditions due to high threshold pH 6. At favorable intestinal conditions, these microparticles dissolved rapidly and the drug released. This diminishes the impact of drug precipitation and improved the absorption. In vivo comparison with drug powder suspension was done by oral dosing in rats and Eudragit L (3 g) loaded microparticles showed twofold increase in bioavailability, although *C*_(max⁡)_ and *T*_(max⁡)_ were not significantly different.

## 6. Conclusion

Despite wide therapeutic applications and worldwide commercialization of cinnarizine, the marketed formulation accounts for low and erratic bioavailability, due to gastric acidity dependent absorption. Progressively, the approach to overcome these limitations had been worked on. Earlier complexation techniques were practiced but now recently developed dosage forms such as SNEDDS, gastroretentive systems, are in interest. Lipid based systems emerged with improved solubility and dissolution in vitro (80–95%) and in vivo bioavailability (AUC approximately 4 times higher than tablets). The purpose to circumvent the obstacles associated with drug was also achieved by the preparations aimed to longer retention at gastric environment. These provided new achievements for sustained release of cinnarizine, so as to reduce dosing frequencies and attain patient compliance. Use of nanosized liquid crystalline particles (cubosomes) can be proved as new field of interest for sustained absorption of cinnarizine.

## 7. Future Perspective

Patient compliance is the ultimate aim for any pharmaceutical dosage form. For the control of drug release or sustained benefits for patients, the control of gastrointestinal transit profile could be the focus of next upcoming time. The microsized particles, microballoons, microspheres, and microsponges alone or in combination with other approaches like use of bioadhesive polymers or coating materials to facilitate intimate adherence to gastric mucosa and incorporation of acidifiers in order to improve pH dependent solubility of cinnarizine, might result in the availability of new product with improved potential for the therapeutics of cinnarizine. Cinnarizine solubility is highly dependent on gastric acidity, so pH controlled systems can be proposed to provide constant rate release, irrespective of location of dosage form in the GIT. Release of cinnarizine from its dosage form independent of environmental conditions can also be achieved by designing osmotically controlled gastroretentive drug delivery systems which can further be modified by coating external surface with some erodible polymer to provide alternate chances of improvement and must be assessed further.

Use of lipid based systems as gastroretentive systems can set forth for the new era of overcoming the pharmacokinetic constraints of cinnarizine. Tablets and capsules formulated using self-emulsifying drug delivery system containing droplets size extending from few nanometers to microns can be developed, while sustaining good flowability, cohesive property, and high content uniformity. Methods by which a transiently supersaturated state can be generated and maintained in situin the GI tract can be set forth for development. In this context, novel formulation approach of use of polymeric precipitation inhibitors or retarders like hydroxyl propyl methyl cellulose incorporated into self-nanoemulsifying delivery systems to produce a supersaturated drug concentration and prolong such a drug concentration for an extended period of time for an optimal absorption, thereby improving oral bioavailability, can be set forth for assessment for future development of cinnarizine. The various techniques discussed herein can be better option for companies to focus on commercializing them. The approach may present a system to potentially improve the pharmacokinetics of the drug.

## Figures and Tables

**Table 1:** A summary of physicochemical, pharmacokinetic and pharmacodynamic features of cinnarizine.

Properties	Pharmacokinetics	Pharmacodynamics
Piperazine derivative	Multiple dosing results in accumulation of drug	Antihistaminic, calcium channel blocker, antidopaminergic, anticholinergic and anti serotinergic.

Weak base	Rapid absorption through upper part of GIT (*T*_max⁡_ = 2–4 h)	Selectively binds to calcium channels in open configuration specifically active in arteries

Poor aqueous solubility (BCS class II)	Cross blood brain barrier by simple diffusion	Blocking effect on L-type calcium channels actively helpful for smooth muscle cells.

pH dependent solubility	Preferably metabolized in liver	Melanogenesis inhibition, regarded as skin whitening agents.

Highly lipophilic (log⁡*P* = 5.71)	Metabolism by oxidation via cytochrome P450	Reduction in membrane permeability to extracellular calcium.

Melting point: 118–122°C	CYP2D6 and CYP2B6 selectively catalyze p-hydroxylation of cinnamyl phenyl ring and diphenyl methyl group respectively.	Exhibits inhibitory effect on potassium currents facilitating effect against vestibular vertigo.

UV (*ƛ*_max⁡_ = 253 nm)	Highly protein bound (91%).	Inhibitory action on 5HT serotonin uptake by platelets.

	Excretion is preferably via urine or in feaces to some extent.	Promote cerebral blood flow.

		Aggravates parkinsonism upon chronic medication.

References to the above mentioned points are cited in the text.

**Table 2:** Clinical therapeutics and trial reports on cinnarizine.

Title	Comments	Reference
Cinnarizine for the prophylaxis of migraine associated vertigo: a retrospective study	After three months of cinnarizine therapy the mean frequency of vertigo and migraine along with the duration and intensity of migraine headaches per month were significantly reduced (*P* < 0.001). The results were suggestive for the safe and effective use of cinnarizine against co-morbidity of migraine and vertigo among patients who suffer from vestibular migraine or migraine with brain stem aura associated with vertigo.	Togha et al., [69]

Comparison of therapeutic effects of alprazolam and cinnarizine in patients with idiopathic tinnitus: a clinical trial study.	Both the drugs were effective for treatment of idiopathic tinnitus. The side effect of drowsiness was less in case of cinnarizine (31.6%) with high rate of partial remission (68.4%).	Salari et al., [70]

Efficacy and safety of cinnarizine in the prophylaxis of migraine headaches in children: an open, randomized comparative trial with propranolol	No significant difference was seen in 50% reduction of the baseline headache frequency between treatment from cinnarizine and propanolol separately (*P* = 0.358) along with insignificant adverse effects. This trial proved cinnarizine to be as effective and safe as propranolol for prophylaxis of migraine in children, but recommended to be confirmed by a double-blind placebo-controlled trial.	Togha et al., [71]

A reassessment of diagnostic criteria and treatment of idiopathic urticarial vasculitis: a retrospective study of 47 patients	Most patients did not show clinical and laboratory features of urticarial vasculitis after treatment with cinnarizine. Thus set forth the use of cinnarizine as effective treatment and suggested as a valuable medication for utricarial vasculatis.	Tosoni et al., [72]

Cinnarizine in refractory migraine prophylaxis: efficacy and tolerability. A comparison with sodium valproate	Cinnarizine and sodium valproate group showed 61.2% and 63.8% subjects as responders towards treatment, respectively with no statistically significant differences, suggesting the efficacy of cinnarizine in severe migraine.	Mansoureh et al., [73]

Open-label trial of cinnarizine in migraine prophylaxis	Significant reduction in attack duration and severity (*P* < 0.001) with no serious adverse events was reported, concluded the efficacy of cinnarizine as well-tolerated prophylactic antimigraine medication with early onset of effectiveness.	Togha et al., [74]

Cinnarizine has an atypical antipsychotic profile in animal models of psychosis.	The drug at a dose of 20 mg/kg significantly counter acted MK-801 (0.25 mg/kg) and amphetamine (5 mg/kg) locomotors effects and remain same on increasing dose to 60 or 180 mg/kg with appearance of mild catalepsy. The report suggested that cinnarizine has a potential antipsychotic effect.	Dall'Igna et al., [75]

Cinnarizine is a useful and well-tolerated drug in the treatment of acquired cold urticaria (ACU)	The report supported the effective and well-tolerated treatment for acquired cold utricaria by administering high dose of drug	Tosoni et al., [76]

New approaches to the management of peripheral vertigo: Efficacy and safety of two calcium antagonists in a 12-week, multinational, double-blind study	Treatment with cinnarizine reduced the incidence of moderate vertigo episodes by 65.8% and extreme vertigo by 89.8%. The report suggested efficacious use of cinnarizine for treatment of peripheral vertigo.	Pianese et al., [77]

Cinnarizine in the prophylaxis of seasickness: laboratory vestibular evaluation and sea study	Effectiveness of cinnarizine on vestibule-ocular reflex supported the potency of cinnarizine for the prevention of seasickness in lab study and during voyage in rough sea.. A dose of 50 mg was resulted in effective medication for prevention of sea sickness.	Shupak et al., [78]

The vestibulo-ocular reflex (VOR) under the influence of cinnarizine	A dose of 25 mg and 50 mg of drug, showed decrease in gained VOR. Reduction was also observed for patients treated with cinnarizine 25 mg in combination with 10 mg domperidone or 1 transdermal scopolamine patch. No notable side effects were observed, suggesting safe use of cinnarizine alone or in combination against VOR.	Doweck et al., [79]

Effect of cinnarizine on various types of vertigo. Clinical and electronystagmographic results of a double-blind study	The study provided favorable results against sudden peripheral vestibular deficit from vertigo of circulatory origin and from post traumatic vertigo. Thus concluded to be well tolerated with minor side effects for treatment against vertigo.	Hausler et al., [80]

Antihypoxidotic and nootropic drugs: Proof of their encephalographic and pharmacodynamic properties by quantitative EEG investigations	The result suggested cinnarizine induced desired EEG changes only in V-EEG showing effectiveness in case of geriatric population.	Saletu and Grunberger, [81]

Cinnarizine in the treatment of chronic asthma	The drug is also shown to exert anti-asthmatic effect in patients with chronic asthma by antagonizing calcium ion transport across the mast cell membrane. The report proved cinnarizine as first drug of a new family of anti-asthmatic drugs offering a protective effect when taken systemically.	Emanuel et al., [82]

**Table 3:** Few brands of Cinnarizine in combination with other drugs.

Brand name	Combination	Manufacturer
Arlevert	Dimenhydrinate	Hampton Pharmaceuticals, UK
Arlevertan	Dimenhydrinate	Hennig Arzneimittel Gmbh & Co., Italy
Azinorm-C	Domperidone	Azine healthcare, India
Cinacris forte	Dihydroergocrocristine	Ivax, Argentina
Cinnasia-D	Domperidone	Willow pharmaceuticals, India
Clinadil	Dihydroergocristine	STADA, Spain
Combitropil	Piracetam	Sintez, Russian Federation
Domstal-CZ	Domperidone	Torrent pharmaceuticals Ltd., India
Exit	Piracetam	Farmasa, Brazil
Neurozine	Paracetamol	Psycardia neurosciencesultramark healthcare Pvt Ltd., India
Omaron	Piracetam	Nizhpharm, Georgia
Phezam	Piracetam	Balkanpharma, Bulgaria
Primatour	Chlorcyclizine	Meda pharma, Neitherland
Rinomar	Phenylpropanolamine	Recip, Sweden
Stugil	Domperidone	J & J (Janssen), India
Vernavo	Domperidone	Neiss labs Pvt. Ltd., India

**Table 4:** Comments on advantages and limitations of cinnarizine delivery systems as included in the text.

Delivery system	Advantages	Limitations
Fast dissolving tablets	Rapid absorption and quick onset of action.	Have insufficient mechanical strength.
	Rapid drug therapy intervention.	Require handling precautions.
	Ease of administration especially to pediatric and geriatric population.	Generally hygroscopic in nature, so require specialized product packaging.
	Disintegrate within seconds without the need of water.	May lead to unpleasant gritty mouth feeling.
	Improve dissolution of poorly soluble drug.	
	Allow high drug loading.	
	Pregastric absorption avoids hepatic metabolism and reduce side effects.	

Lipid based systems	Enhance solubilization of poorly soluble drugs.	Lack of sound in vitro predictive models.
	Overcome dissolution step.	Chemical instability of drugs.
	Submicron droplets size increases surface area for absorption resulted in increased rate and extent of absorption.	Need high surfactant concentration (30–60%).
	Selectively target to specific site in GIT.	May permit less drug loading.
	More consistent drug absorption.	Lack of appropriate in vitro-in vivo correlation.
	Resist precipitation of drug in upper part of GIT and on shifting the pH, compatible for cinnarizine.	May exhibit limited lymphatic uptake from emulsion based systems.
	Unsaturated fatty acids (an essential excipient) enhance solubility.	Oxidation of unsaturated fatty acids in the formulation.
	Prevent drug degradation in GIT.	
	Diminishes fasted and fed state variation in absorption and also food effect.	
	Liquid crystalline nanostructured particles provided better scope for sustained delivery.	
	Cubosomes improvise stability, bioadhesivity and biocompatibility.	
	Hexasomes accentuate stability of encapsulated drug	

Gastroretentive systems	Residence in stomach for longer duration provides sustained effect.	Floating tablets and films undergo all or none effects.
	Suitable system for drugs that have absorption window in upper GIT.	May be swept away due to Migrating Myoelectric Complex motility pattern
	Diminishes precipitation of basic drug at alkaline pH.	May cause gastric irritation.
	Appreciable therapeutic activity.	Bioadhesive systems have high turnover rate of mucus.
	Account for once a day therapy.	Require presence of food to delay gastric emptying.
	Advantageous for drugs with narrow therapeutic index.	Microspheres, microballoons have low drug loading capacity.
	Lesser risk of dose dumping for multiparticulate systems.	
	Less inter and intra subject variability.	
	Microspheres, microballoons provide high degree of dispersion in digestive tract.	

References to the above mentioned points are cited in the text.

## References

[1] Kumar P., Singh C. (2013). A study on solubility enhancement methods for poorly water soluble drugs. *The American Journal of Pharmacological Sciences*.

[2] Chen H., Khemtong C., Yang X., Chang X., Gao J. (2011). Nanonization strategies for poorly water-soluble drugs. *Drug Discovery Today*.

[3] Gu C.-H., Rao D., Gandhi R. B., Hilden J., Raghavan K. (2005). Using a novel multicompartment dissolution system to predict the effect of gastric pH on the oral absorption of weak bases with poor intrinsic solubility. *Journal of Pharmaceutical Sciences*.

[4] Fagerberg J. H., Tsinman O., Sun N., Tsinman K., Avdeef A., Bergström C. A. S. (2010). Dissolution rate and apparent solubility of poorly soluble drugs in biorelevant dissolution media. *Molecular Pharmaceutics*.

[5] Patel B., Jayvadan P., Rashmin T. (2010). Improvement of solubility of cinnarizine by using solid dispersion technique. *International Research Journal of Pharmacy*.

[6] Li B.-Q., Yang G.-Q., Fang S.-H., Gao J.-Y., Gu F.-M., Dong X., Zhang J.-X., Wang Y. (2010). Effect of route of administration on the pharmacokinetics and toxicokinetics of cinnarizine in dogs. *European Journal of Pharmaceutical Sciences*.

[7] Kornhuber J., Henkel A. W., Groemer T. W., Städtler S., Welzel O., Tripal P., Rotter A., Bleich S., Trapp S. (2010). Lipophilic cationic drugs increase the permeability of lysosomal membranes in a cell culture system. *Journal of Cellular Physiology*.

[8] Kariya S., Isozaki S., Narimatsu S., Suzuki T. (1992). Oxidative metabolism of cinnarizine in rat liver microsomes. *Biochemical Pharmacology*.

[9] Kariya S., Isozaki S., Uchino K., Suzuki T., Narimatsu S. (1996). Oxidative metabolism of flunarizine and cinnarizine by microsomes from B-lymphoblastoid cell lines expressing human cytochrome P450 enzymes. *Biological and Pharmaceutical Bulletin*.

[10] Narimatsu S., Kariya S., Isozaki S., Ohmori S., Kitada M., Hosokawa S., Masubuchi Y., Suzuki T. (1993). Involvement of CYP2D6 in oxidative metabolism of cinnarizine and flunarizine in human liver microsomes. *Biochemical and Biophysical Research Communications*.

[11] Sweetman S. C. (2002). *Martindle: The Complete Drug Reference*.

[12] Turner D., Lurie Y., Finkelstein Y., Schmid T., Gopher A., Kleid D., Bentur Y. (2006). Pediatric cinnarizine overdose and toxicokinetics. *Pediatrics*.

[13] López M. G., Moro M. A., Castillo C. F., Artalejo C. R., Garcia A. G. (1989). Variable, voltage-dependent, blocking effects of nitrendipine, verapamil, diltiazem, cinnarizine and cadmium on adrenomedullary secretion. *British Journal of Pharmacology*.

[14] Broekaert A., Godfraind T. (1979). A comparison of the inhibitory effect of cinnarizine and papaverine on the noradrenaline- and calcium-evoked contraction of isolated rabbit aorta and mesenteric arteries. *European Journal of Pharmacology*.

[15] Shi S., Chen H., Cui Y., Tang X. (2009). Formulation, stability and degradation kinetics of intravenous cinnarizine lipid emulsion. *International Journal of Pharmaceutics*.

[16] Haasler T., Homann G., Duong Dinh T. A., Jüngling E., Westhofen M., Lückhoff A. (2009). Pharmacological modulation of transmitter release by inhibition of pressure-dependent potassium currents in vestibular hair cells. *Naunyn-Schmiedeberg's Archives of Pharmacology*.

[17] Pukhal'skaya T. G., Kolosova O. A., Men'shikov M. Y., Vein A. M. (2000). Effects of calcium antagonists on serotonin-dependent aggregation and serotonin transport in platelets of patients with migraine. *Bulletin of Experimental Biology and Medicine*.

[18] Medeiros Y. S., Calixto J. B. (1991). Influence of calcium entry blockers and calmodulin inhibitors on 5-hydroxytryptamine-, potassium- and calcium-induced contractions in human umbilical artery in-vitro. *Journal of Pharmacy and Pharmacology*.

[19] Kariya S., Isozaki S., Masubuchi Y., Suzuki T., Narimatsu S. (1995). Possible pharmacokinetic and pharmacodynamic factors affecting parkinsonism inducement by cinnarizine and flunarizine. *Biochemical Pharmacology*.

[20] Chang T.-S., Lin V. C.-H. (2011). Melanogenesis inhibitory activity of two generic drugs: cinnarizine and trazodone in mouse B16 melanoma cells. *International Journal of Molecular Sciences*.

[21] Marti Masso J. F., Obeso J. A., Carrera N., Martinez-Lage J. M. (1987). Aggravation of Parkinson's disease by cinnarizine. *Journal of Neurology Neurosurgery and Psychiatry*.

[22] http://www.drugs.com/international/cinnarizine.html.

[23] http://www.drugsupdate.com/brand/showavailablebrands/467.

[24] Ogata H., Aoyagi N., Kaniwa N., Ejima A., Sekine N., Kitamura M., Inoue Y. (1986). Gastric acidity dependent bioavailability of cinnarizine from two commercial capsules in healthy volunteers. *International Journal of Pharmaceutics*.

[25] Shahba A. A.-W., Mohsin K., Alanazi F. K. (2012). Novel self-nanoemulsifying drug delivery systems (SNEDDS) for oral delivery of cinnarizine: design, optimization, and in-vitro assessment. *AAPS PharmSciTech*.

[26] Burton P. S., Goodwin J. T., Vidmar T. J., Amore B. M. (2002). Predicting drug absorption: how nature made it a difficult problem. *Journal of Pharmacology and Experimental Therapeutics*.

[27] Tokumura T., Ueda H., Tsushima Y., Kasai M., Kayano M., Amada I., Machida Y., Nagai T. (1984). Inclusion complex of cinnarizine with *β*-cyclodextrin in aqueous solution and in solid state. *Journal of Inclusion Phenomena*.

[28] Tokumura T., Nanba M., Tsushima Y., Tatsuishi K., Kayano M., Machida Y., Nagai T. (1986). Enhancement of bioavailability of cinnarizine from its *β*-cyclodextrin complex on oral administration with DL-phenylalanine as a competing agent. *Journal of Pharmaceutical Sciences*.

[29] Tokumura T., Tsushima Y., Tatsuishi K., Kayano M., Machida Y., Nagai T. (1986). Enhancement of the bioavailability of cinnarizine from its *β*-cyclodextrin complex on oral administration with L-isoleucine as a competing agent. *Chemical and Pharmaceutical Bulletin*.

[30] Tokumura T., Tsushima Y., Tatsuishi K., Kayano M., Machida Y., Nagai T. (1987). Enhancement of the oral bioavailability of cinnarizine in oleic acid in beagle dogs. *Journal of Pharmaceutical Sciences*.

[31] Järvinen T., Järvinen K., Schwarting N., Stella V. J. (1995). *β*-Cyclodextrin derivatives, SBE4-*β*-CD and HP-*β*-CD, increase the oral bioavailability of cinnarizine in beagle dogs. *Journal of Pharmaceutical Sciences*.

[32] Kalava B. S., Demirel M., Yazan Y. (2005). Physicochemical characterization and dissolution properties of cinnarizine solid dispersion. *Turkish Journal of Pharmaceutical Sciences*.

[33] Mehta D. M., Parejiya P. B., Barot B. S., Shelat P. K. (2012). Investigation of the drug release modulating effect of acidifiers in modified release oral formulation of cinnarizine. *Asian Journal of Pharmaceutical Sciences*.

[34] Patel R. P., Suthar A. (2009). Formulation and process optimization of cinnarizine fast-release tablets. *Pharmaceutical Technology*.

[35] Nagar M., Yadav A. V. (2009). Cinnarizine orodispersible tablets: a chitosan based fast mouth dissolving technology. *International Journal of PharmTech Research*.

[36] Basu B., Bagadiya A., Makwana S., Vipul V., Batt D., Dharamsi A. (2011). Formulation and evaluation of fast dissolving tablets of cinnarizine using superdisintegrant blends and subliming material. *Journal of Advanced Pharmaceutical Technology and Research*.

[37] Raghavendra Rao N. G., Kulkarni U., Patil B. S. (2011). Design and development of fast dissolving Cinnarizine tablets by sublimation technique. *Research Journal of Pharmacy and Technology*.

[38] Patel B. P., Patel J. K., Rajput G. C., Thakor R. S. (2010). Formulation and evaluation of mouth dissolving tablets of cinnarizine. *Indian Journal of Pharmaceutical Sciences*.

[39] Heer D., Aggarwal G., Kumar S. L. H. (2014). Development of fast dissolving oral films and tablets of cinnarizine: effect of superdisintegrants. *International Journal of Pharmacy and Pharmaceutical Sciences*.

[40] Thomas N., Holm R., Rades T., Müllertz A. (2012). Characterising lipid lipolysis and its implication in lipid-based formulation development. *The AAPS Journal*.

[41] Nanjwade B. K., Patel D. J., Udhani R. A., Manvi F. V. (2011). Functions of lipids for enhancement of oral bioavailability of poorly water-soluble drugs. *Scientia Pharmaceutica*.

[42] Lee K. W., Porter C. J., Boyd B. J. (2013). The effect of administered dose of lipid-based formulations on the In Vitro and In Vivo performance of cinnarizine as a model poorly water-soluble drug. *Journal of Pharmaceutical Sciences*.

[43] Larsen A. T., Ogbonna A., Abu-Rmaileh R., Abrahamsson B., Østergaard J., Müllertz A. (2012). SNEDDS containing poorly water soluble cinnarizine; development and *In vitro* characterization of dispersion, digestion and solubilization. *Pharmaceutics*.

[44] Larsen A. T., Ohlsson A. G., Polentarutti B., Barker R. A., Phillips A. R., Abu-Rmaileh R., Dickinson P. A., Abrahamsson B., Østergaard J., Müllertz A. (2013). Oral bioavailability of cinnarizine in dogs: relation to SNEDDS droplet size, drug solubility and in vitro precipitation. *European Journal of Pharmaceutical Sciences*.

[45] Larsen A. T., Åkesson P., Juréus A., Saaby L., Abu-Rmaileh R., Abrahamsson B., Østergaard J., Müllertz A. (2013). Bioavailability of cinnarizine in dogs: effect of SNEDDS loading level and correlation with cinnarizine solubilization during in vitro lipolysis. *Pharmaceutical Research*.

[46] Boyd B. J., Khoo S.-M., Whittaker D. V., Davey G., Porter C. J. H. (2007). A lipid-based liquid crystalline matrix that provides sustained release and enhanced oral bioavailability for a model poorly water soluble drug in rats. *International Journal of Pharmaceutics*.

[47] Vithlani S., Sarraf S., Chaw C. S. (2012). Formulation and in vitro evaluation of self-emulsifying formulations of Cinnarizine. *Drug Development and Industrial Pharmacy*.

[48] Lee K. W. Y., Porter C. J. H., Boyd B. J. (2013). Gastric pre-processing is an important determinant of the ability of medium-chain lipid solution formulations to enhance oral bioavailability in rats. *Journal of Pharmaceutical Sciences*.

[49] Shi S., Chen H., Lin X., Tang X. (2010). Pharmacokinetics, tissue distribution and safety of cinnarizine delivered in lipid emulsion. *International Journal of Pharmaceutics*.

[50] Yasir M., Rai S. (2012). Cinnarizine loaded lipid based system: preparation, optimization and in-vitro evaluation. *IOSR Journal of Pharmacy*.

[51] Sassene P. J., Knopp M. M., Hesselkilde J. Z., Koradia V., Larsen A., Rades T., MüLlertz A. (2010). Precipitation of a poorly soluble model drug during in vitro lipolysis: characterization and dissolution of the precipitate. *Journal of Pharmaceutical Sciences*.

[52] Christiansen M. L., Holm R., Kristensen J., Kreilgaard M., Jacobsen J., Abrahamsson B., Müllertz A. (2014). Cinnarizine food-effects in beagle dogs can be avoided by administration in a Self Nano Emulsifying Drug Delivery System (SNEDDS). *European Journal of Pharmaceutical Sciences*.

[53] Christophersen P. C., Christiansen M. L., Holm R., Kristensen J., Jacobsen J., Abrahamsson B., Müllertz A. (2014). Fed and fasted state gastro-intestinal in vitro lipolysis: in vitro in vivo relations of a conventional tablet, a SNEDDS and a solidified SNEDDS. *European Journal of Pharmaceutical Sciences*.

[54] Kayaert P., Anné M., van den Mooter G. (2011). Bead layering as a process to stabilize nanosuspensions: influence of drug hydrophobicity on nanocrystal reagglomeration following in-vitro release from sugar beads. *Journal of Pharmacy and Pharmacology*.

[55] Shanmugam T., Banerjee R. (2011). Nanostructured self assembled lipid materials for drug delivery and tissue engineering. *Therapeutic Delivery*.

[56] Kossena G. A., Charman W. N., Boyd B. J., Porter C. J. H. (2004). A novel cubic phase of medium chain lipid origin for the delivery of poorly water soluble drugs. *Journal of Controlled Release*.

[57] Nguyen T.-H., Hanley T., Porter C. J. H., Boyd B. J. (2011). Nanostructured liquid crystalline particles provide long duration sustained-release effect for a poorly water soluble drug after oral administration. *Journal of Controlled Release*.

[58] Nguyen T.-H., Hanley T., Porter C. J. H., Boyd B. J. (2011). Nanostructured reverse hexagonal liquid crystals sustain plasma concentrations for a poorly water-soluble drug after oral administration. *Drug Delivery and Translational Research*.

[59] Machida Y., Inouye K., Tokumura T., Iwata M., Nagai T. (1989). Preparation and evaluation of intragastric buoyant preparations. *Drug Design and Delivery*.

[60] Varshosaz J., Tabbakhian M., Zahrooni M. (2007). Development and characterization of floating microballoons for oral delivery of cinnarizine by a factorial design. *Journal of Microencapsulation*.

[61] Nagarwal R. C., Ridhurkar D. N., Pandit J. K. (2010). In vitro release kinetics and bioavailability of gastroretentive cinnarizine hydrochloride tablet. *AAPS Pharmaceutical Sciences and Technology*.

[62] Tarkase K. N., Tarkase M. K., Dokhe M. D., Wagh V. S. (2012). Formulation, optimization and comparative evaluation of Gastroretentive drug delivery system of Cinnarizine and Domperidone maleate floating tablet. *International Journal of Pharmaceutical Sciences Review and Research*.

[63] Patnaik A., Mantry S. (2012). Formulation & evaluation of gastroretensive floating microsphere of CINNARiZINE. *Asian Journal of Pharmaceutical and Clinical Research*.

[64] Kumar P., Bhatia M. (2010). Functionalization of chitosan/methylcellulose interpenetrating polymer network microspheres for gastroretentive application using central composite design. *PDA Journal of Pharmaceutical Science and Technology*.

[65] Patel J. K., Patil P. S., Sutariya V. B. (2013). Formulation and characterization of mucoadhesive microparticles of cinnarizine hydrochloride using supercritical fluid technique. *Current drug delivery*.

[66] Singh I., Rana V. (2013). Iron oxide induced enhancement of mucoadhesive potential of Eudragit RLPO: formulation, evaluation and optimization of mucoadhesive drug delivery system. *Expert Opinion on Drug Delivery*.

[67] Verma S., Nagpal K., Singh S. K., Mishra D. N. (2014). Unfolding type gastroretentive film of cinnarizine based on ethyl cellulose and hydroxypropylmethyl cellulose. *International Journal of Biological Macromolecules*.

[68] Alhnan M. A., Murdan S., Basit A. W. (2011). Encapsulation of poorly soluble basic drugs into enteric microparticles: a novel approach to enhance their oral bioavailability. *International Journal of Pharmaceutics*.

[69] Togha M., Taghdiri F., Jahromi S. R., Refaeian F. (2014). Cinnarizine for the prophylaxis of migraine associated vertigo: a retrospective study. *SpringerPlus*.

[70] Salari R., Hatefi H., Abbasi E., Soltani-Gerdefaramarzi H.-R., Mortazavi M.-H. (2013). Comparison of therapeutic effects of alprazolam and cinnarizine in patients with idiopathic tinnitus: a clinical trial study. *Journal of Mazandaran University of Medical Sciences*.

[71] Togha M., Malamiri R. A., Rashidi-Ranjbar N., Asa S., Mahvelati F., Ashrafi M. R. (2012). Efficacy and safety of cinnarizine in the prophylaxis of migraine headaches in children: an open, randomized comparative trial with propranolol. *Acta Neurologica Belgica*.

[72] Tosoni C., Lodi-Rizzini F., Cinquini M., Pasolini G., Venturini M., Sinico R. A., Calzavara-Pinton P. (2009). A reassessment of diagnostic criteria and treatment of idiopathic urticarial vasculitis: a retrospective study of 47 patients. *Clinical and Experimental Dermatology*.

[73] Mansoureh T., Rahmat Jirde M., Nilavari K., Ashrafian H., Razeghi S., Kohan L. (2008). Cinnarizine in refractory migraine prophylaxis: efficacy and tolerability. A comparison with sodium valproate. *Journal of Headache and Pain*.

[74] Togha M., Ashrafian H., Tajik P. (2006). Open-label trial of cinnarizine in migraine prophylaxis. *Headache*.

[75] Dall'Igna O. P., Tort A. B. L., Souza D. O., Lara D. R. (2005). Cinnarizine has an atypical antipsychotic profile in animal models of psychosis. *Journal of Psychopharmacology*.

[76] Tosoni C., Lodi-Rizzini F., Bettoni L., Toniati P., Zane C., Capezzera R., Venturini M., Calzavara-Pinton P. (2003). Cinnarizine is a useful and well-tolerated drug in the treatment of acquired cold urticaria (ACU). *European Journal of Dermatology*.

[77] Pianese C. P., Hidalgo L. O. V., González R. H., Madrid C. E., Ponce J. E. C., Ramírez A. M., Morán L. M., Arenas J. E. P., Rubio A. T. Y. G., Uribe J. O., Abiuso J., Hanuch E., Alegría J., Volpi C., Flaskamp R., Sanjuán A. P., Gómez J. M. G., Hernández J., Pedraza A., Quijano D., Martínez C., Castañeda J. R., Guerra O. J. C., F G. V. (2002). New approaches to the management of peripheral vertigo: efficacy and safety of two calcium antagonists in a 12-week, multinational, double-blind study. *Otology and Neurotology*.

[78] Shupak A., Doweck I., Gordon C. R., Spitzer O. (1994). Cinnarizine in the prophylaxis of seasickness: laboratory vestibular evaluation and sea study. *Clinical Pharmacology and Therapeutics*.

[79] Doweck I., Gordon C. R., Spitzer O., Melamed Y., Shupak A. (1994). The vestibulo-ocular reflex (VOR) under the influence of cinnarizine. *Journal of Vestibular Research: Equilibrium and Orientation*.

[80] Hausler R., Sabani E., Rohr M. (1989). Effect of cinnarizine on various types of vertigo. Clinical and electronystagmographic results of a double-blind study. *Acta Oto-Rhino-Laryngologica Belgica*.

[81] Saletu B., Grunberger J. (1980). Antihypoxidotic and nootropic drugs: proof of their encephalotropic and pharmacodynamic properties by quantitative EEG investigations. *Progress in Neuro-Psychopharmacology*.

[82] Emanuel M. B., Chamberlain J. A., Whiting S., Rigden B. G., Craven A. H. (1979). Cinnarizine in the treatment of chronic asthma. *British Journal of Clinical Pharmacology*.

